# The Production Processes and Biological Effects of Intravenous Immunoglobulin

**DOI:** 10.3390/biom6010015

**Published:** 2016-03-09

**Authors:** Ana Filipa Barahona Afonso, Cristina Maria Pires João

**Affiliations:** 1 Department of Chemistry, Universidade de Évora, Colégio Luís António Verney, Rua Romão Ramalho 59, 7000-671 Évora, Portugal; filipabafonso@gmail.com; 2 Hematology Department, Champalimaud Center for the Unknown, Av. Brasília, 1400-038 Lisboa, Portugal

**Keywords:** IVIg, immunoglobulin, immune modulation, molecular mechanisms, IVIg production

## Abstract

Immunoglobulin is a highly diverse autologous molecule able to influence immunity in different physiological and diseased situations. Its effect may be visible both in terms of development and function of B and T lymphocytes. Polyclonal immunoglobulin may be used as therapy in many diseases in different circumstances such as primary and secondary hypogammaglobulinemia, recurrent infections, polyneuropathies, cancer, after allogeneic transplantation in the presence of infections and/or GVHD. However, recent studies have broadened the possible uses of polyclonal immunoglobulin showing that it can stimulate certain sub-populations of T cells with effects on T cell proliferation, survival and function in situations of lymphopenia. These results present a novel and considerable impact of intravenous immunoglobulin (IVIg) treatment in situations of severe lymphopenia, a situation that can occur in cancer patients after chemo and radiotherapy treatments. In this review paper the established and experimental role of polyclonal immunoglobulin will be presented and discussed as well as the manufacturing processes involved in their production.

## 1. Introduction

In 1890 von Behring firstly communicated their work in serum application against diphtheria [1] and tetanus [2]. Since then, intravenous immunoglobulin (IVIg) has been object of intense investigation in terms of function, clinical application, molecular structure and its modification, and purification.

Human immunoglobulin G (IgG) has been used to treat people with inherited immunoglobulin deficiencies since 1952 when Bruton infused it in a child with undetectable “gamma globulin” levels and who suffered from recurrent pneumococcal infections [3]. Subcutaneous infusions of gamma globulin at monthly intervals induced serum measurable gamma globulin levels and completely eliminated pneumococcal infections. Human IgG soon became the standard treatment for patients with primary antibody deficiencies who develop chronic bacterial infections [3]. Later, in 1981, the use of IVIg for the treatment of autoimmune diseases was first described [4]. Nowadays, IVIg is the major product on the global blood product market and its consumption is multiplying every year in North America, Europe and Asia for licensed and unlicensed/off-label uses.

The active substances in IVIg preparations are polyclonal natural antibodies synthesized, in response to immune stimuli (antigens and T cells), by plasma B cells.

Immunoglobulins are a group of closely related glycoproteins composed of 82%–96% protein and 4%–18% carbohydrate. These glycoproteins of about 150 kDa are present in plasma at a mean concentration of 7 to 12 g/L depending upon individual variations and level of environmental exposure to antigens. The immunoglobulin G (IgG), a major effector molecule of the humoral immune response in man accounts for about 75% of the total Igs in the plasma of healthy individuals. The Igs of the other classes (IgM, IgA, IgD and IgE) each of which has specific properties and functions, constitute the other 25% of the Igs [5].

The basic Ig molecule has a four-chain structure, comprising two identical heavy (H) chains and two identical light (L) chains, linked together by inter-chain disulfide bonds. Intra-chain disulfide bonds are responsible for the formation of loops, leading to the compact domain-like structure of the molecule. The amino terminal portions of the H and L chains, characterized by a highly variable (V) amino acid composition, are referred to a V_H_ and V_L_, respectively. The constant part (C) of the L chain is designated as CL, while that of the H chain are further divided into three distinct subunits: C_H_1, C_H_2 and C_H_3. IgG possesses dual functions characterized by the capacity to recognize and react specifically with the antigen and to perform a series of non-specific effector functions in which the antigen is rendered harmless and eventually eliminated. This functional dichotomy of IgG is reflected in the structure of the molecule that comprises two variable regions responsible for antigen binding (Fab), and a constant region (the Fc or crystallisable fragment) that mediates the specific effector functions.

The Fab fragments are composed of a light chain and part of a heavy chain, whereas the Fc fragment has various regions (C_H_), which exhibit several functions such as binding of complement component 1 (C1) and interactions with the Fc receptor on macrophages or neutrophils and other immune cells. The activation of the effector function is initiated by an aggregation of the IgG molecule on the surface of the antigen. Such activation exposes molecular structures that can activate the complement system or induce opsonization by phagocytes [6]. Immunoglobulins, B and T cells are the key mediators of adaptive immunity. Deficiencies in either of these two arms of immunity can lead to a heightened susceptibility to bacterial, fungal or viral infections. Primary immunodeficiency (PID) disorders, such as agammaglobulinaemias, hyperimmunoglobulin M (IgM) syndromes and common variable immunodeficiencies (CVID), are either caused by defined gene mutations or remain molecularly undefined [7,8]. In addition, hypogammaglobulinaemic phenotypes, termed secondary immunodeficiencies, can arise for example from viral infections, B cell malignancies, bone marrow transplantation or after immunosuppressive therapy [7]. For most of the primary and secondary Ig deficiencies, intravenous Ig replacement therapy (IVIg) is the treatment of choice.

The polypeptide chains of Igs are encoded by three non-linked cluster of autosomal genes, one cluster coding for H chains of all classes and subclasses, a second one for kappa (k) light chains and a third one for lambda (λ) light chains. These three genes clusters are called the H-, k-and λ gene families respectively. In humans the H gene family is on chromosome 14, the k gene family is on chromosome 2 and the λ gene family is on chromosome 22. Molecular genetic studies have revealed the arrangement of gene segments within the H chain and L chain families. Each H chain is encoded by 4 distinct types of gene segments, designated V_H_ (variable), D_H_ (diversity), J_H_ (joining) and C_H_. The V region of the H chain is encoded by the V_H_, D_H_ and J_H_ segments. The L chains are encoded by the 3 gene segments, V_L_, J_L_ and C_L_ segments.

The C gene segments of the H and L chains encode for the constant regions. Nine immunoglobulin H chain isotypes are found in humans: IgM, IgE, IgG (with subclasses IgG1, IgG2, IgG3 and IgG4) and IgA (with subclasses IgA1 and IgA2).

The C_H_ gene segments determine the class and/or subclass of the H chain, whereas V_H_, D_H_ and J_H_ regions determine the antigen-recognizing part of the Ig molecule. The H and L chains constant genes lie 3′ to the V_H_, D_H_, J_H_, and V_L_, J_L_ genes, respectively. During maturation of progenitor B cells to mature B cells an active H chain exon is formed by V_H_, D_H_ and J_H_, and that of L chain formed by V_L_ and J_L_ somatic gene rearrangements (recombined V_H_D_H_J_H_ and V_L_J_L_) which codes for antigen binding variable region of IgG, followed by linkage to a certain C_H_ gene locus. This is transcribed to mRNA and subsequently translated to an immunoglobulin H chain molecule. The C_H_ gene closest to the J_H_ locus is the Cμ gene (IgM), which is the first isotype gene to be expressed. The other C_H_ genes can subsequently be expressed by downstream switching mechanisms with simultaneous deletion of the original isotypic C_H_ genes. The DNA rearrangements that underlie isotype switching and confer their functional diversity on the humoral immune response are directed by cytokines, especially those released by effector CD4 T cells [9].

## 2. IVIg’s Composition and Industrial Production Methods

Intravenous immunoglobulins (IVIgs) are a therapeutic preparation of pooled normal polyspecific human IgGs obtained from large numbers of healthy donors. The preparation contains antibodies to microbial antigens, self antigens (natural autoantibodies) and anti-idiotypic antibodies which recognize other antibodies [10]. These categories are not mutually exclusive [11].

Plasma used in the production of IVIg comes from two origins: approximately 20 percent is from blood donors, and the other 80 percent is from plasma donors [12]. Individual plasmas are pooled; the pool size is a minimum of 1000 donors, but may be up to 100,000 donors [5,13,14,15]. The maximum number of donors in pools is treated as proprietary information by each manufacturer. The many thousands of donors who contribute to a typical pool of plasma used for isolation of immunoglobulin represent a wide range of antibody specificities against infectious agents [13,15] such as bacterial, viral and also a large number of self antigens reflecting the cumulative exposure of the donor population to the environment.

Techniques developed by Cohn and his coworkers [16,17] in the USA at the beginning of World War II led to the development of the separation of plasma proteins into individual stable fractions with different biologic functions. The basis for Cohn’s fractionation was to use low concentrations of alcohol, reducing the pH and lowering ionic strength. The procedure was performed at low temperature, which reduced the likelihood of contamination and made large-scale fractionation possible. This method, further refined in cooperation with J. L. Oncley [17], is basically still in use and, with some additional steps, yields Ig for intravenous and subcutaneous use [6].

The products are licensed as 50 mg/mL (5%) or 100 mg/mL (10%) IgG solutions for infusion, but may vary slightly from lot to lot and significantly from manufacturer to manufacturer [18].

Preparations of IVIg consist of intact IgG molecules with a distribution of IgG subclasses corresponding to that of normal serum. Subclass distribution may vary between preparations, with some products having less than physiological levels of IgG3 and/or IgG4. IVIg also contains small, but variable, amounts of other proteins and products, notably, and depending on the commercial preparation, albumin, IgA (content varying from less than 5 μg/mL to more than 700 μg/mL), IgE, IgM, sugars, salts, trace amounts of solvents, detergents and buffers may contribute to tolerability difficulties [19]. The monomer and/or dimer content may vary between preparations and up to 3% non-active polymers may be found. Several of these proteins and products may affect the tolerability of IVIg infusions, notably salt, sugar and/or IgA content; volume, pH, osmolality and rate of infusion. The half-life of IVIg after intravenous infusion or intramuscular injection is approximately 2–3 weeks. This can, however, vary depending on the immune status of the patient.

After blood collection (and posterior separation of cellular components) or plasma collection, the plasma is typically stored at ≤ −20°C for several months. All donations are screened for virus infections (Hepatitis B, Hepatitis C and Human immunodeficiency virus) [20,21]. A specified acceptable maximum titre of ABO blood groups antibodies is present, reducing the risks of haemolytic reactions due to the presence of ABO antibodies or antibodies to other blood group system in IVIg [22]. The quality of each individual plasma donation through using good collection practices is crucial to optimize pathogen safety and to ensure the preservation of protein functional activities. Collecting plasma for fractionation are inspected by National Regulatory Authorities (NRAs) and audited by fractionators. In Europe, details on plasma collection procedures, testing, handling and transportation are assembled into a “Plasma Master File” (PMF) [5,21,23].

Recently, the manufacturing processes have much evolved ensuring good *in vivo* tolerance and minimizing side effects, in particular transmission of infectious agents and improved recovery [5,24]. The manufacturers have strived to develop processes which do not treat the proteins as harshly as reducing and alkylating. These gentler processes result in benefits to the patients [24]:

- Improved or increased efficacy;
- Shorter and easier to manage processes will increase consistency;
- More efficient processes will increase supply;
- Increased purity leads to better tolerability and efficacy;
- An optimized formulation, such as a liquid increases convenience and may improve tolerability.

Here we describe the primary purification processes for commercial IVIg production.

### 2.1. Fractionation

The alteration of proteins solubility in various salt solutions and the theoretical relationship between solubility of proteins and ionic strength led to the application of fractionation by organic solvents to serum proteins. In 1946, his principle was applied by Cohn and coworkers establishing a 5 variable system for the separation of albumin plasma protein based on the addition of ethanol, pH variability, conductivity, protein concentration and temperature [16]. Summarily, the five most abundant proteins were enriched in Fractions I to V by sequential precipitation by increasing concentration of ethanol: fibrinogen in Fraction I, γ-globulins in Fraction II, lipid-bearing β-globulins in Fraction III, the α-globulins in Fraction IV and albumin in Fraction V.

After Deutsch and colleagues (1946) increased the yield of IgG in Fraction II by lowering the ionic strength for precipitation of Fraction III [25]. In 1949, Oncley’s separation method of Fraction III from Fraction II + III, was improved based on the work of Deutsch *et al.* A detailed separation into 4 sub-fractions III containing isoagglutinins (III-1), prothrombin (III-2), plasminogen (III-3) and certain lipoproteins (III-0) led to a satisfactory separation of Fraction II containing only antibodies [17,26]. Cohn used filtration technology instead of centrifugation for the removal of Fraction III, improving IgG yield using this same method [27].

Fraction II + III from Cohn’s method has become the starting material for the isolation of most IVIg processes [20,24]. The Cohn–Oncley process is still the dominant method for the production of IVIg utilized by most companies today with minor modifications [28].

The Kistler–Nitschmann process, a variation of the cold ethanol precipitation, was developed in the 1950s by the Central Laboratory of the Swiss Red Cross (today ZLB-Behring, a subsidiary of CSL Limited) for a supposedly better yield with less manipulation [29]. This process is only utilized today by ZLB-Behring and their licensees.

In 1962, Kistler and Nitschmann changed the fractionation scheme of IgG by reducing the ethanol content from 17% to 12% for the precipitation of precipitate B (corresponding to Fraction (I + III) [29]. In return the yield of IgG was increased at the expense of the purity. The IgG containing precipitate contains besides 90% γ-globulins also 2% β-globulins and 8% albumin. The different precipitation stages must be performed below freezing point to avoid denaturing of the proteins. This is considered the main disadvantage of ethanol fractionation technology in addition to the quite low yield. This process is currently used by ZLB-Behring and their licensees.

In the 1960s it was shown that short fatty acids (C6-C12) form insoluble complexes with α- and β-globulins whereas γ-globulins are not as readily precipitated [30]. Steinbuch and co-workers described a purification process for IgG with caprylate (*i.e.*, octanoate, a C8-saturated fatty acid) as precipitating agent [31]. Non-immunoglobulins were precipitated from human plasma after dilution with an acetate buffer to reach a final pH of 4.8. After addition of caprylate under vigorous stirring, an IgG enriched solution was obtained. The purity and yield depended on the amount of caprylic acid, the pH, the molarity of the buffer and the dilution factor. Later, in 2003, Lebing selected Cohn–Oncley Fraction II + III as starting material for the isolation of IVIg by two caprylate precipitation steps combined with 2 anionic exchange steps [32]. The precipitate, which contains lipoproteins, albumin, other plasma proteins, and some caprylic acid, is removed by cloth filtration in the presence of filter aid, a procedure that also removes many potential viruses [20].

Polyethylene glycol (PEG) is a synthetic polymer that is also used to separate proteins by fractional precipitation from a natural mixture like plasma by an exclusion mechanism. The solubility of plasma components in the presence of variable concentrations of PEG 6000 at different pHs varies in accordance with their electrophoretic mobility [33]. This method isolates albumin and γ-globulin from plasma, in which IgG is precipitated from plasma by 13% PEG 6000. After treatment with a cationic exchanger, re-precipitation, batch adsorption to an anionic exchanger and precipitation of IgG by 25% ethanol a pure IgG product is yielded [34].

### 2.2. Chromatography

Purification of immunoglobulins by ion-exchange chromatography on diethylaminoethyl (DEAE) cellulose columns was reported in 1959 [35]. DEAE chemical groups bear a positive charge and bind to ions (anions) and proteins that have an overall negative charge.

In the 60’s, IgG was separated from human serum in a 2-step batch procedure using DEAE-Sephadex [36]. 97% pure IgG could be recovered in the supernatant. Composed of cross-linked dextran beads with DEAE chemical groups attached, DEAE Sephadex^®^ had uniform particle size and high protein binding capacity. DEAE Sephadex^®^ column chromatography was used to produce highly purified IgG from small volumes of human plasma in a one-step purification process. IgG recovery was approximately 95% [37]. The success of ion-exchange chromatography in purifying proteins led to development of improved ion-exchange resins. In 1985, Friesen *et al.* reported purification of human IgG using the cross-linked agarose gels DEAE-Sepharose and DEAE-Biogel [38].

Although the new generation of resins has an improved binding capacity [39] large columns are still needed. Large buffer volumes have to be applied to completely elute the IgG from the column. The IgG fraction is highly diluted, resulting in large volumes.

More recent method prepares the IgG more or less directly from plasma and not from a precipitate. Lipoproteins are removed from plasma or Cohn I supernatant (of fraction I) by the treatment of colloidal silica to prevent column fouling. Two anion exchange chromatography steps are performed in the flow through mode. First the IgG containing solution is loaded to a DEAE Sepharose FF column at pH 5.2 and secondly a macroporous anion-exchange resin at pH 6.5 is used. Virus inactivation methods pasteurization and treatment at low pH are integrated in the process [40].

Hydrophobic Charge Induction Chromatography (HCIC) was created in the 90’s by Ciphergen Biosystems Ltd. for the purification of antibodies. This form of chromatography was first described by Burton and Harding [41]. The technique is based on the pH dependent behaviour of an ionisable dual mode ligand. Binding is based on hydrophobic interaction and molecular recognition of the ligand, elution is based on the electrostatic charge-repulsion due to the change of charge of the ligand at a different pH. Two different media are available, the first one, MEP HyperCel, carries a 4-Mercapto-Ethyl-Pyrdine ligand, and the second MBI HyperCel, carries a 2-mercapto-5-benzimidazole sulfonic acid ligand. MEP binds antibodies in a pH range from 7 to 9 and elutes antibodies at a pH of 4, whereas MBI binds antibodies in a pH range from 5 to 5.5 and elutes them at a pH of 8.8 to 9.5. A study performed by Buchacher and Iberer showed that purity of the eluate was not satisfactory, still huge amounts of fibrinogen, IgA and IgM were present [20].

Size exclusion chromatography is suited for the final phase of the separation process and allows the separation of the different IgG forms according to their respective sizes. Thus IgG solutions can be separated in poly-, di- and monomers under mild conditions. IVIg preparations prepared from pooled plasma of thousands of healthy donors contain monomeric and dimeric IgG, whereas IVIg isolated from one donor contains only IgG monomers [42].

### 2.3. Virus Inactivation and Removal

IVIg is made up of plasma pools of thousands of donors; this represents a substantial risk for viral transmission. HIV and HCV are notorious transmissible viruses, but a good level of purity is found since IVIg fractionation steps result in the removal of a significant portion of viruses [43]. Nevertheless, manufacturers take into account 3 measures: a careful selection of donors, careful screening of donated units for known infective agents and the use of fractionation methods, which include specific steps designed to remove or inactivate viruses [44].

The available methods for virus inactivation and removal include solvent/detergent (S/D) treatment. The inactivation of most enveloped viruses is very fast, typically achieved within the first 15 min. This time depends on the mixture of S/D reagents, the conditions and the matrix of the solution. After the inactivation step the S/D-reagents have to be removed from the product [45]. The caprylate virus inactivation in which a lipophilic non-ionized caprylate disrupts viral lipid envelopes and renders the virus non-infectious [32,46]. Ionic disruption of the envelope and conformational changes of viral proteins are needed for docking the virus to the target cell, *i.e.*, by low pH incubation [47]. Pasteurization, the heat treatment of IVIg solutions at 60 °C for 10 h is used by some manufacturers [48]. UVC radiation treatment is developing as a complementary virus inactivation method preferentially for non-enveloped small viruses. Low dose-radiation may destroy virus, attacking specifically the nucleic acids, while proteins remain intact [49]. Nanofiltration is a special kind of ultrafiltration, as the product can pass the membrane, while viruses are retained [50]. A very effective removal of viruses in the purification scheme is accomplished by precipitation, especially when the precipitate is a waste fraction. This is also the most effective step to remove prions [51]. Different types of chromatography are also used to remove virus with several physiochemical and biochemical properties [52]. The implementation of chromatographic procedures also aims at the removal of IgA, a cause of severe anaphylactic shock in patients with IgA deficiency presenting anti-IgA antibodies. Also, the ratios of sub-types of IgG are similar to its physiological proportions.

Neutralizing antibodies against e.g., HAV, Polio-1 and Parvovirus B19 contribute significantly to the virus safety of IVIg preparations. Such antibodies are present in plasma pools and are concentrated during purification of IVIg [53].

## 3. Pharmacokinetics of IVIg

Human normal immunoglobulin is immediately and completely bioavailable in the recipient’s circulation after intravenous administration. It is distributed relatively rapidly between plasma and extravascular fluid, after approximately 3–5 days equilibrium is reached between the intra and extravascular compartments.

The half-lives of most IgG antibodies are considerably longer, 6–8 days in mice [54] and 7–21 days in healthy humans [55] and 33–36 days in immunodeficient patients receiving IVIg infusions [56]. This increases the availability of sufficient specific IgG to fight infection.

After administration of relatively large amounts of IVIg (0.1–2 g/kg body mass), the IgG concentration in serum immediately rises, falls rapidly in the first 1 to 7 days, and then falls more slowly thereafter. The initial rapid fall is associated with passage of IgG out of the vasculature into lymph and extracellular fluid compartments. The subsequent decline is mainly caused by catabolism while IgG in lymph and tissues slowly diffuses back into the circulation [57].

The median serum half-life of IgG is 23 days, which is much longer than for IgM (5.1 days) and IgA (5.6 days) [57]. In the early 1960s, Brambell and colleagues proposed that for IgG to have such a long half-life there must be a receptor that binds IgG and prevents its catabolism (the *Brambell hypothesis*) [58]. Furthermore, these authors proposed that, with low levels of IgG would be less competition for binding to the protective receptor (FcRp) and the half-life of IgG would be longer; with high levels of IgG would be more competition for FcRp and the half-life for IgG would be shorter. Further studies by Brambell *et al.* demonstrated that FcRn, the gut Fc receptor responsible for transport of maternal immunoglobulin from ingested milk to the systemic circulation, had characteristics similar to those hypothesized for FcRp. In 1996 two groups simultaneously established that FcRp and FcRn are the same receptor [59,60]. We now know that the long serum half-life of IgG is attributed to this neonatal Fc receptor, named FcRn, which is composed of a MHC class I-related protein and a b2-microgloblin. IVIg preparations have a similar blood half-life to endogenous immunoglobulin, thus monthly replacement therapy is usually adequate. However, the half-life of IgG can be abnormally short in certain conditions, e.g., protein-losing enteropathy, nephrotic syndrome, IgG paraproteinemia, and myotonic dystrophy. On the contrary, patients with hypogammaglobulinemia have a prolonged serum half-life of IgG and, conversely, administering high doses of IVIg accelerates IgG catabolism and shortens the half-life of IgG [13].

Table 1 summarizes the pharmacokinetic parameters of IVIg.

## 4. Mechanisms of Action of IVIg

IVIg has an immunomodulatory activity that is based on the modulation of biological processes that are implicated in innate or acquired immunity.

The biological effects of IVIg include:

### 4.1. Functional Blockade of Fc Receptors

Fc receptors, present on phagocytes, are implicated in the clearance of particles or cells that were opsonized by IgG. This process is of great physiological importance in the mechanisms of defense against infectious agents. Also, this process may contribute to the pathogenesis of certain auto-antibody-mediated diseases. This is the case in peripheral autoimmune cytopenias (e.g., idiopathic thrombocytopenic purpura). In such situations, the saturation of Fc receptors by IVIg leads to decreased cellular destruction as a consequence of Fc-mediated phagocytosis of antibody-coated cells [19,62,63].

### 4.2. Autoantibody Neutralization and Inhibition of Autoantibody Production

IVIg preparations contain anti-idiotypic antibodies, *i.e.*, antibodies that are able to interact specifically with the variable region (antigen recognition site) of autoantibodies. This interaction has the potential to neutralize an autoantibody and to hamper its production via binding to auto reactive B lymphocytes. Such a neutralizing or inhibitory activity has been shown, amongst others, for autoantibodies directed against factor VIII, DNA, intrinsic factor, thyroglobulin and anti neutrophil cytoplasmic antibodies (ANCA) [64,65].

### 4.3. Complement Inhibition

The Fc portion of IVIg can bind the C3b and C4b fragments of complement, and thereby inhibit their tissue deposition as well as the generation of the C5 convertase (C4b2a3b), hampering the subsequent formation of the membrane attack complex [66]. This cascade of events has been shown to occur in dermatomyositis and thereby contributes to the therapeutic effect of IVIg in that disease.

### 4.4. Modulation of Cytokine and Cytokine Antagonist Production

IVIg may significantly modulate the production of several cytokines (including IL-1, -2, -3, -4, -5, -10, TNF-α, and GM-CSF) and cytokine antagonists (IL-1 receptor antagonist) by monocyte-macrophages and lymphocytes [67]. Although quite complex, it appears that the resulting biological anti-inflammatory effects implicate both the F(ab)2 and Fc portions of IgG, are associated with an inhibition of lymphocyte proliferative responses, and a modulation of T helper cytokine production [19].

### 4.5. Activation or Functional Blockade of the Death Receptor Fas (CD95)

It has been shown that IVIg can either inhibit or activate cell death by binding to the death receptor Fas [68]. IVIg contains IgGs that bind to several distinct epitopes on the extra cellular portion of Fas and are also naturally occurring anti-Fas antibodies. It has been shown that these can be divided into agonistic anti-Fas IgG and antagonistic anti-Fas IgG [69]. IVIg preparations contain both agonistic and antagonistic anti-Fas antibodies and either trigger or protect from cell death. This process is unclear at present, but appears in part to depend on the cell type concerned.

### 4.6. Modulation of Dendritic Cell Properties

Recent work suggests that IVIg also affects the differentiation, maturation and functional status of dendritic cells (DCs). DCs appear to be a primary target for the immunosuppressive effects of IVIg on T-cell activation. This effect of IVIg is mediated notably by inhibition of the differentiation and maturation of DCs, with down-regulation of the expression of co-stimulatory molecules, thus impairing the ability of mature DCs to produce IL-12, and enhancing their ability to produce IL-10 [70,71].

### 4.7. Signaling through the Inhibitory Fc Receptor, Fcgamma RIIB

The molecular basis of anti-inflammatory properties of IVIg has been investigated in a murine model of immune thrombocytopenia by Samuelsson *et al.* [63]. In this model, administration of clinically protective doses of intact antibody or monomeric Fc fragments prevented platelet consumption triggered by a pathogenic auto-antibody. The inhibitory Fc receptor, Fcgamma RIIB, was shown to be required for this protection, as disruption either by genetic deletion or with a blocking monoclonal antibody, reversed the therapeutic effect of IVIg. IVIg were also shown to induce the expression of this inhibitory receptor on monocytes.

### 4.8. Enhanced Steroid Sensitivity

Work by Spahn and colleagues [72] in asthmatic patients has shown that the action of IVIg, which is used as an oral glucocorticoid-sparing agent in patients with steroid-dependent asthma, is due to an enhancement in glucocorticoid receptor-binding affinity, subsequent enhanced glucocorticoid sensitivity, and synergistic suppression of lymphocyte activation when combined with glucocorticoids. In an open-label clinical study, IVIg was shown to result in significant reductions in oral glucocorticoid dose, and both patients with glucocorticoid-insensitive and -sensitive asthma responded equally well to IVIg [72].

### 4.9. Expand Lymphocyte Repertoire Diversity

Recent data demonstrate that IVIg may have a role on broaden T cell repertoire diversity in situations where oligloclonality is present as autoimmune disorders [73,74]. The study of the effect of IVIg therapy on TCR repertoires in SLE patients, found that their perturbation was relatively reduced under clinically successful IVIg therapy, supporting the notion that the beneficial effect of IVIg includes a stabilization of diversified T. The finding that repertoire diversity under IVIg treatment was significantly correlated with individual Treg surface CD25, thus likely with Treg activity, does not exclude Treg-independent effects of IVIg that could also inhibit specific clonal expansion. So, it seems that under IVIg therapy, Tregs may substantially contribute to controlling and limiting expanded T-cell clones, likely including pathogenic ones. Also, B cell repertoire may be affected by IVIg treatment as reported by others [75]. The self molecules of polyclonal immunoglobulin seem to have a crucial role on the promotion of T cell development and function, both acting on the thymus as well as in the periphery [76,77,78].

## 5. Utilization of IVIg in Human Disorders and Immune Reconstitution

Despite controversy about the benefit on the use of poly specific immunoglobulin in the context of infections after stem cell transplantation, this agent has been made part of transplantation units’ protocols since long ago. However, in 2003, Cordonnier and colleagues, published the results of a randomized, double blind, dose effect and placebo controlled multicenter trial with the objective of assessing the role and dose effect relationship of immunoglobulin in the prophylaxis of complications after allogeneic stem cell transplantation. The results do not recommend the use of prophylactic immunoglobulin in allogeneic recipients of stem cell transplant from HLA-identical sibling donors [79]. Nevertheless, several other studies had showed benefit regarding a lower incidence of infectious episodes during IVIg prophylaxis [80,81].

Polyclonal immunoglobulin, acting as an immune activator has been used, in the context of research, to promote lymphocyte development and also to improve lymphocyte function. Several studies have been describing how IVIg improve T cell receptor repertoire diversity in the thymus and periphery and also how it expands T cell receptor repertoire and induce naïve T cell proliferation [76,78,82]. Old evidence in experimental animals and in man indicates that normal immunoglobulin selects pre-immune B cell repertoires in the bone-marrow and peripheral lymphoid tissues [83,84]. This essential immunomodulatory role of IVIg is believed to be dependent of the content on natural autoantibodies presented in the preparations of IVIg [85]. Several roles are defended for these natural antibodies as defense against infections, clearance of aging cells, role in the process of antigen presentation of T cells, anti-tumoral surveillance, anti-inflammatory activity, and selection of immune repertoires and homestasis of auto reactivity [86].

Various uses of IVIg in human disorders are known. Here, we summarize the main uses in Table 2.

## 6. Adverse Effects of IVIg Treatment

The management of the adverse effects of IVIg is symptomatic, and in view of their mildness, they rarely require any aggressive treatment with the exception of uncommon anaphylactic reactions or cardiovascular or respiratory complications (Table 3). Adverse reactions to intravenous immune globulin occur in less than 5 percent of patients [62,70]. Mild reactions to immune globulin occur within the first 30 min after infusion and may be relieved by reducing the infusion rate or temporarily stopping the infusion [87]. Adverse reactions are particularly likely in a patient who has not received IVIg previously and who has or recently has had a bacterial infection [88]. Thus vigilance needs to be maintained for detecting and managing reactions, irrespective of an individual patient’s personal experience with IVIg. Inter product pharmaceutical differences (*i.e.*, osmolality, sugar content, and IgA content) should be considered in relation to the patient’s clinical and physiologic status. IgG tends to form aggregates which are believed to be responsible for the majority of the adverse effects. Methods as light scattering should be used additionally to size exclusion chromatography during the optimization of the process trying to decrease infusion related adverse effects [89,90].

## 7. Conclusions

IVIg has saved and improved the quality of life of patients with a variety of immunodeficiency diseases and conditions responsive to immunomodulation. However, the widening array of off-label conditions for which this product is administered cries out for more systematic evaluations of efficacy. This need is especially critical because, despite an overall excellent safety record, IVIG can cause serious complications.

The immunomodulatory effects of IVIg depend on both the administered dose and the disease under investigation. In spite of many uncertainties, high-dose IVIG is presently a valuable treatment option for a number of autoimmune diseases. A better understanding of pathogenesis of many inflammatory diseases is also needed to be able to study the effect of IVIG in the relevant clinical conditions.

There is increasing understanding that the mechanisms can be effective concomitantly and synergistically depending on the circumstances it is used, but studies are mainly speculative confirming the urgent need of clinical research in both mechanisms involved. Additional investigations are required to understand which of the mechanisms are more prominent under given *in vivo* conditions. 

## Figures and Tables

**Table 1:** Pharmacokinetic parameters of commercialized IVIg in Primary Immunodeficiency Diseases and Secondary Immunodeficiency Diseases [61].

	Level of Evidence	IVIg Preparation	Dose (g/kg)	Clearance (mL/kg/day) ^(a)^	Volume of Distribution (L/kg) ^(a)^	Half-Life (days) ^(a)^
Primary Immunodeficiency	II-2	Intraglobin	0.1–0.346	1.8–2.4	0.051–0.059	33–43
		Gamimune	0.1–0.225	—	—	22
		Gamimune	0.5	—	—	~14
		Sandoglobulin	0.4	—	—	31–32
		SNBTS IV IgG	0.2–0.54	—	—	15–53
		Sandoglobulin	0.1–0.4	—	—	32
		Gammagard	0.4	—	—	32
		Venogamma	0.163–0.671	—	—	12
		Flebogamma	0.3–0.6	0.52–0.89	0.028	37
		Gamimune N	0.3–0.4	—	—	35
Secondary Immunodeficiency Diseases	Bone marrow transplantation					
	I	Gammagard	0.25 or 0.5	11–14.6	0.1–0.134	6
	II-2	Gammagard	0.5	—	—	6.1
	II-2	Sandoglobulin	0.5	—	—	5.6
	II-2	Gamimune-N	0.5	—	—	1.3–1.9
	II-2	Supplied by The Netherlands Red Cross	0.2	—	—	1.3–2.8 ^(b)^
	Chronic lymphocytic leukemia, non-Hodgkin’s lymphoma, or multiple myeloma					
	II-2	Gamimune	0.15–0.5	—	—	7–20
	II-2	Gammagard	0.4	—	—	39
	Burn injuries					
	II-2	Gamimune	0.5	—	—	Initial (day 0–1): 4.4
				—	—	Terminal (day 1–4): 12.4
	I	Sandoglobulin	0.5	—	—	Within 3 days of injury: 47 h
				—	—	In third postburn wk: 154 h

**^(a)^** Data are mean or range of means; ^(b)^ Cytomegalovirus-specific immunoglobulin G.

**Table 2:** List of IVIg uses.

**Licensed Indications**
Primary immunodeficiency [91,92,93];
Wiskott Aldrich syndrome [94,95];
IgG subclass deficiencies with recurrent infections [96,97];
Idiopathic Thrombocytopenic Purpura [98,99,100];
Kawasaki disease [101,102];
Common variable immunodeficiency [103,104];
Multiple myeloma/CLL [105];
Children with HIV [106];
Guillain-Barre syndrome [107,108];
Allogenic bone marrow transplantation—prevention of graft *versus* host disease (GVHD) and infections [109]
**Clinical evidence: Unlicensed Neurological Conditions**
Acute disseminated encephalomyelitis [110,111,112,113,114];
Chronic inflammatory demyelinating poly-radiculoneuropathy [115,116];
Dermatomyositis or polymyositis [117,118,119,120,121];
Inclusion body myositis [122,123];
Lambert-Eaton myasthenic syndrome [124];
Multifocal motor neuropathy [125];
Multiple sclerosis [126,127];
Myasthenia gravis [128,129];
Neuromyotonia [130,131];
Other peripheral neuropathies [132,133,134,135];
Paraneoplastic disorders: encephalomyelitis [136,137], limbic encephalitis [138], cerebellar degeneration [139], peripheral neuropathy [125,132,140] and opsoclonus-myoclonus syndrome [141];
Paraprotein associated demyelinating neuropathy [142,143];
Stiff Man Syndrome [144];
Vascular disorders of the CNS: small vessel vasculitis [145], renal lupus [146,147] and antiphospholipid syndrome [148,149]
**Clinical evidence: Unlicensed uses—others non neurological situations**
Systemic vasculitis [150,151,152,153];
Systemic lupus erythematosus [154];
Burns septicaemia [155];
Rheumatoid arthritis [156];
Nephrotic syndrome [157];
Septic shock [158,159,160,161];
Toxic shock syndrome [162];
Clostridium difficile diarrhea [163,164]

**Table 3:** Adverse reactions to IVIg.

Adverse Reactions	Minor ^(a)^	Moderate ^(b)^	Serious ^(c)^
Infusion reactions	Nausea	Vomiting	
	Low grade fever		
	Feeling sick		
	Dizziness		
	Chest tightness		
	Fatigue		
	Rigor		
Cardiovascular	Tachycardia	Hypotension	Apoplexy
	Flushing		Stroke
	Chills		Cardiac arrest
	Headache		Myocardial infarction
	Migraine		Deep venous thrombosis
	Palpitation		Thromboembolic reaction
			Vascular collapse
Renal events			Acute Renal failure
Neurological	Anxiety	Chest tightness	Coma
	Irritability	Loss of consciousness	shock
	Nervousness		
	Seizures		
	Tremor		
Hematologic			Haemolysis
			Pancytopenia leukopenia
			positive direct antiglobulin (Coombs) test
Respiratory	Cough	Apnea,	Pulmonary embolus
		Wheezing	Pulmonary edema
		Dyspneia	Acute Respiratory Distress Syndrome (ARDS),
		Cyanosis,	Transfusion Associated Lung Injury (TRALI)
		Hypoxemia,	
		Bronchospasm	
Dermatological	Facial flush	Eczema	Steven-Johnson syndrome, epidermolysis,
	Skin rash/urticaria		erythema multiforme,
			bullous dermatitis
Musculoskeletal	Low backache		
	Myalgia		
Inflammation			Aseptic meningitis
Gastrointestinal	Abdominal pain	Hepatic dysfunction	
Rheumatologic	Arthralgias		
Allergic reaction			Anaphylaxis (associated with sensitization to IgA in patients with IgA deficiency)

**^(a)^** They are generally rate related, and diminish rapidly on reducing the rate of infusion; **^(b)^** May necessitate discontinuation of the infusion and the administration of hydrocortisone and an antihistamine; **^(c)^** According to International Conference on Harmonization of Technical Requirements for Registration of Pharmaceuticals for Human Use (ICH) Guidelines: any untoward medical occurrence that at any dose: results in death, is life-threatening, requires inpatient hospitalization, or prolongation of existing hospitalization, results in persistent or significant disability/incapacity, or is a congenital anomaly/birth defect. Serious adverse reactions are more frequently related with increasing immunoglobulin levels (high doses IVIg). Importance of early identification of risk factors associated with serious adverse reactions to IVIg.
